# Ovarian seromucinous tumors: clinicopathological features of 10 cases with a detailed review of the literature

**DOI:** 10.1186/s13048-021-00796-y

**Published:** 2021-03-18

**Authors:** Romana Idrees, Nasir Ud Din, Sabeehudin Siddique, Saira Fatima, Jamshid Abdul-Ghafar, Zubair Ahmad

**Affiliations:** 1 Department of Pathology and Laboratory Medicine, Aga Khan University Hospital, Karachi, Pakistan; 2 Department of Pathology and Clinical Laboratory, French Medical Institute for Mothers and Children (FMIC), Kabul, Afghanistan

**Keywords:** Borderline tumor, Concomitant ovarian tumor, Endometriosis, Stromal invasion, Seromucinous carcinoma, Endometrioid carcinoma with mucinous differentiation

## Abstract

**Background:**

The 2014 WHO Classification of ovarian neoplasms introduced a new entity of seromucinous tumors associated with endometriosis. These tumors encompassed a spectrum from benign to malignant and included seromucinous cystadenoma/ cystadenofibroma, seromucinous borderline tumor/atypical proliferative seromucinous tumor and seromucinous carcinoma. However, the 2020 WHO Classification of Female Genital Tumours removed seromucinous carcinomas as a distinct entity and recategorized them as Endometrioid carcinomas with mucinous differentiation. Here we describe clinico-morphologic features of seromucinous tumors recategorizing cases originally diagnosed as seromucinous carcinoma in light of 2020 WHO classification and present detailed review of literature.

**Methods:**

**S**lides of seromucinous tumors were reviewed. Special emphasis was given to evaluation of stromal invasion. Follow-up was obtained.

**Results:**

Ten cases were diagnosed. Mean age was 40 years. Four cases were bilateral. Mean size was 19 cm. Grossly; luminal papillary projections were seen in 6 cases. Tumors demonstrated a papillary architecture with papillae lined by stratified seromucinous epithelium showing nuclear atypia. Stromal invasion was seen in 4 cases. Six cases were reported as borderline seromucinous tumors and 4 cases originally diagnosed as seromucinous carcinoma were recategorized as endometrioid carcinoma with mucinous differentiation on review. Endometriosis was seen in 4 cases. CK7, PAX8 and ER were positive in 7/7 cases. Two cases showed extra-ovarian involvement. Follow up was available in 7 cases. Six patients were alive and well at follow up ranging from 8 to 46 months. Six patients received chemotherapy postoperatively. One patient with carcinoma died of disease 18 months postoperatively.

**Conclusion:**

In our series, 4 cases were originally diagnosed as seromucinous carcinomas. However, these were recategorized in light of the 2020 WHO Classification of Female Genital tumors as endometrioid carcinomas with mucinous differentiation. Six cases were diagnosed as seromucinous borderline tumors. Thus, majority of cases were borderline in agreement with published literature.

## Background

The 4th edition World Health Organization (WHO) classification of Tumors of Female Reproductive Organs published in 2014 made a number of modifications in the classification of ovarian tumors especially epithelial tumors, modified the grading system for serous carcinomas. It also identified a new morphological group of seromucinous tumors which are believed to be derived from or associated with endometriosis in a number of cases. These tumors in the 2014 classification encompassed a spectrum from benign to malignant and included seromucinous cystadenoma/cystadenofibroma, seromucinous borderline tumor/atypical proliferative seromucinous tumor and seromucinous carcinoma. These tumors are morphologically composed of serous and mucinous (endocervical type) epithelium. Foci composed of transitional, squamous, clear cell or endometrioid epithelium are sometimes seen. These tumors were first described in 2002. However, the 5th edition of WHO Classification of Female Genital Tumors published in 2020 has removed seromucinous carcinoma as a distinct entity and now considers it as a subtype of endometrioid carcinoma. In 2012, the International Federation of Gynecology and Obstetrics (FIGO) Oncology Committee revised the FIGO Classification for staging of ovarian, tubal and primary peritoneal cancers and the new FIGO Staging Classification was published and became effective in early 2014 [1–6]. We had diagnosed 10 cases of ovarian seromucinous tumors in our practice since their inclusion in the 2014 WHO classification as a distinct entity. Herein, we aim to discuss the clinicopathological features of these 10 cases and the recategorization of the cases originally diagnosed as seromucinous carcinoma in the light of the new 2020 WHO Classification of Female Genital Tumours. We believe that this is the first series of seromucinous ovarian tumors from South Asia. We also present a detailed review of the published literature on these rare tumors.

## Methods

The Surgical Pathology files of the Section of Histopathology were searched for all cases of ovarian tumors diagnosed as seromucinous tumors. All cases diagnosed as seromucinous cystadenoma, seromucinous borderline tumor or seromucinous carcinoma between January 1, 2015 and December 31, 2019 were included in the study. Clinical features of all diagnosed cases were recorded. Hematoxylin and Eosin (H&E) slides of all cases were retrieved and blindly reviewed by the two principal authors (RI and ZA). Clinical and pathological features of all cases were described. All cases were carefully evaluated for stromal invasion. Special stain Periodic acid–Schiff (PAS) ± Alcian Blue (AB) was used to highlight the intracellular acid mucin. Immunohistochemical (IHC) stains for Cytokeratin (CK) 7 (monoclonal mouse anti human, ready to use, Glostrup, Dako, Denmark), and PAX8 (anti-PAX8, mouse monoclonal primary antibody, Cell Marque, Rocklin, CA, USA) were used to demonstrate expression in epithelial cells. CK20 (monoclonal mouse anti human, ready to use, Glostrup, Dako, Denmark), Estrogen Receptor (ER) (monoclonal rabbit anti human, clone EP1, ready to use, Glostrup, Dako, Denmark), Progesterone Receptor (monoclonal anti human, clone PgR636, ready to use, Glostrup, Dako, Denmark), Wilms Tumor 1 (WT1) (monoclonal mouse, anti- human, ready to use, Glostrup, Dako, Denmark), and CDX2 (monoclonal mouse anti-human, ready to use, Glostrup, Dako, Denmark) were performed in selected cases. Clinical follow up was obtained from the patients or family members.

## Results

A total of 10 seromucinous ovarian tumors were reported during the study period. Details of clinicopathological features are shown in Table 1. Ages of the patients ranged from 25 to 57 years with mean and median age of 40 and 41 years, respectively. The tumors were bilateral in 4 cases (40%), left ovary alone was involved in 2 cases (20%) while right ovary alone was involved in 3 cases (30%). In 1 case, laterality was not known. Symptoms included abdominal pain, swelling and distension, bleeding per vagina etc. and were present in 9 cases for several months before the patients sought medical attention. One patient, however, had a short two-day history of severe abdominal pain following cyst rupture and secondary peritonitis. Magnetic Resonance Imaging (MRI) and ultrasound revealed large cystic masses in the ovaries. The cysts were multi-septate and uni or multilocular.

**Table 1:** Clinicopathological data of cases in our series (*n* = 10)

S.No	Age (years)	Laterality	Size (cm)	Histological diagnosis
1	41	Left	7x7x3	Endometriod carcinoma with mucinous differentiation
2	25	Not Known	35 × 34	Borderline seromucinous tumor
3	32	Right	11.5 × 10.5	Borderline seromucinous tumor
4	50	Bilateral	Right 25 × 29 Left 10 × 6	Borderline Seromucinous tumor
5	54	Not Known	Not Known	Seromucinous carcinoma & Adult granulosa cell tumor
6	34	Not Known	23 × 13.5	Borderline seromucinous tumor
7	36	Left	6x4x4	Borderline seromucinous tumor
8	57	Bilateral	Left 10 × 7 Right 10 × 9.5	Borderline seromucinous tumor
9	50	Right	18 × 12	Seromucinous carcinoma
10	53	Right	12 × 5	Seromucinous carcinoma

Hysterectomy with bilateral salpingo-oophorectomy was performed in 6 cases (60%), while ovarian cystectomy was performed in 4 cases (40%). On gross examination, sizes of cysts ranged from 7 cm to 35 cm with mean size of 19 cm. Cysts were bi or multilocular in 7 cases (70%) and unilocular in 3 cases (30%).

**Fig. 1:**
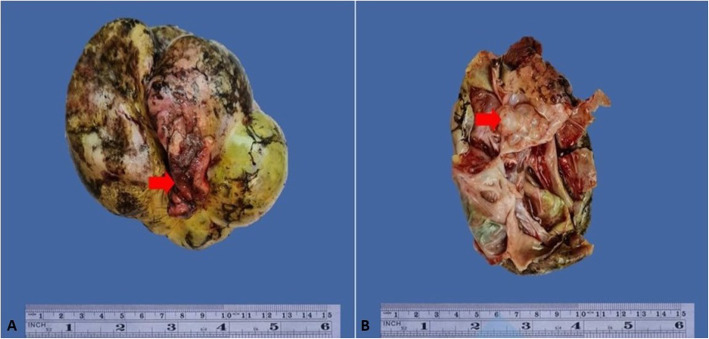
**a** Picture of an ovarian seromucinous borderline tumour. The capsule is intact with smooth outer surface. Note the attached fallopian tube (→). **b** On opening the cyst shows multiple locules (→) that were filled with thick myxoidy material

External surfaces of cysts were smooth in 9 cases (90%) **(**Fig. 1a). On opening, the cysts were filled with light brown to hemorrhagic myxoidy material **(**Fig. 1b). In 1 case, the external surface showed cauliflower like projections. In 6 cases (60%), inner surfaces of cysts demonstrated multiple papillary excrescences or projections.

**Fig. 2:**
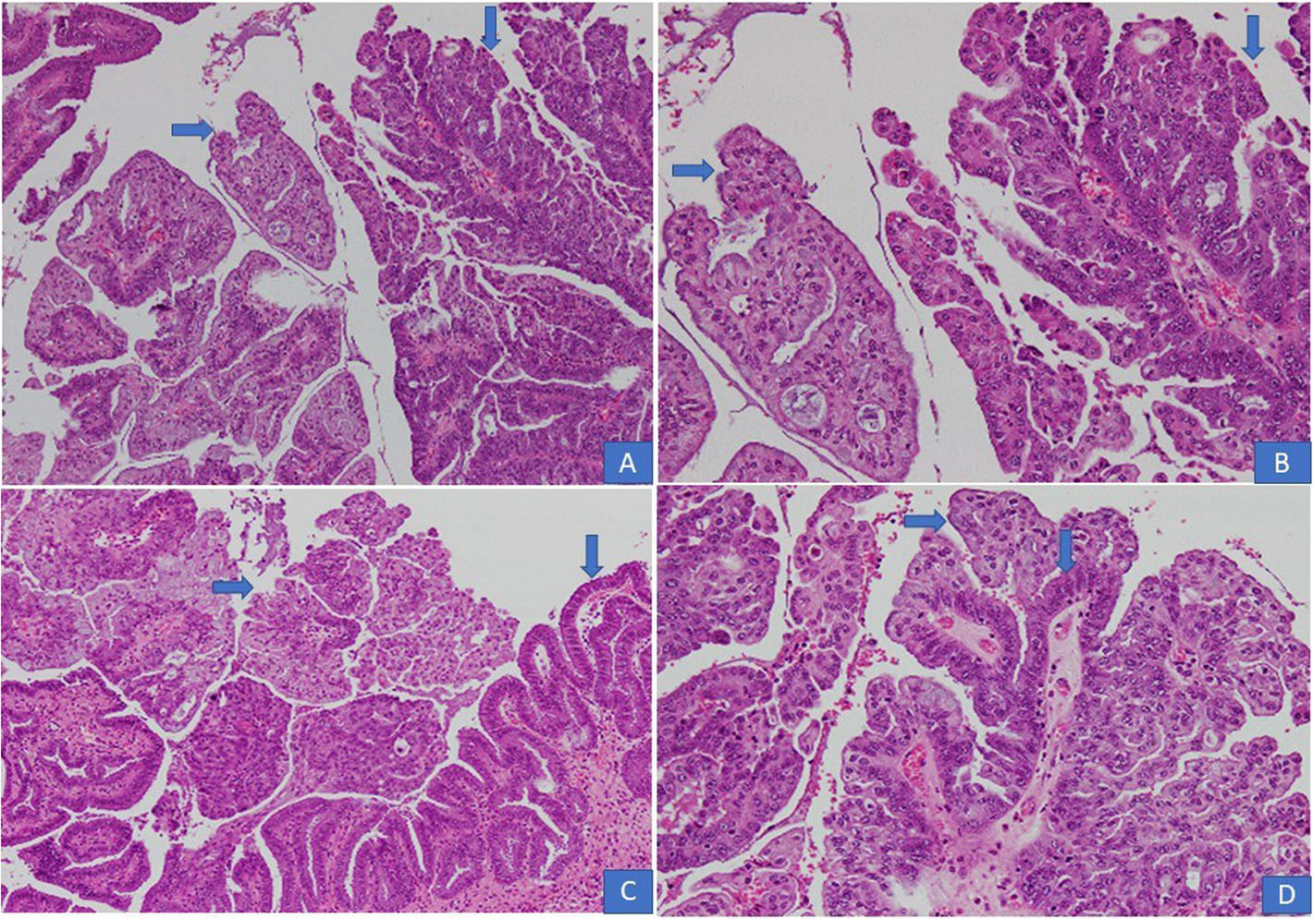
**a**-**d**. Seromucinous borderline tumor. Papillae with fibrovascular cores lined by stratified epithelium. Both serous (↓) and mucinous (→) epithelium in different areas of the tumor (H&E × 20)

**Fig. 3:**
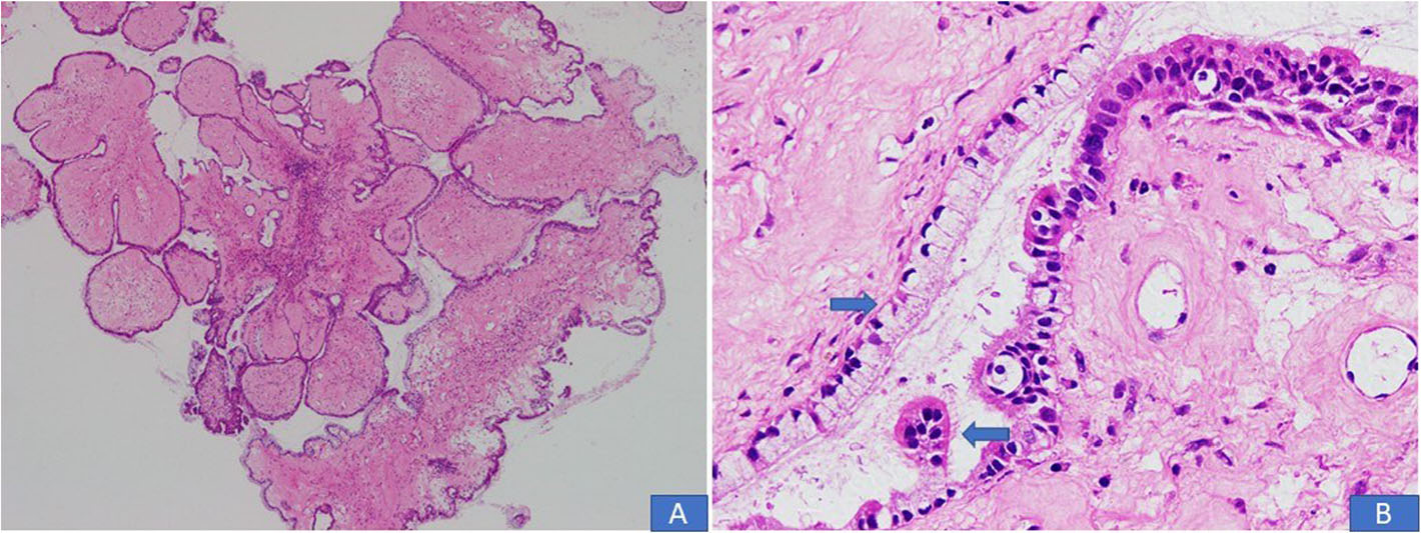
**a** Most of the seromucinous borderline tumors had fibromatous stroma. **b** both endocervical type mucinous columnar epithelium (→) and ciliated serous epithelium (↓) are seen in the same field (H&E × 20)

**Fig. 4:**
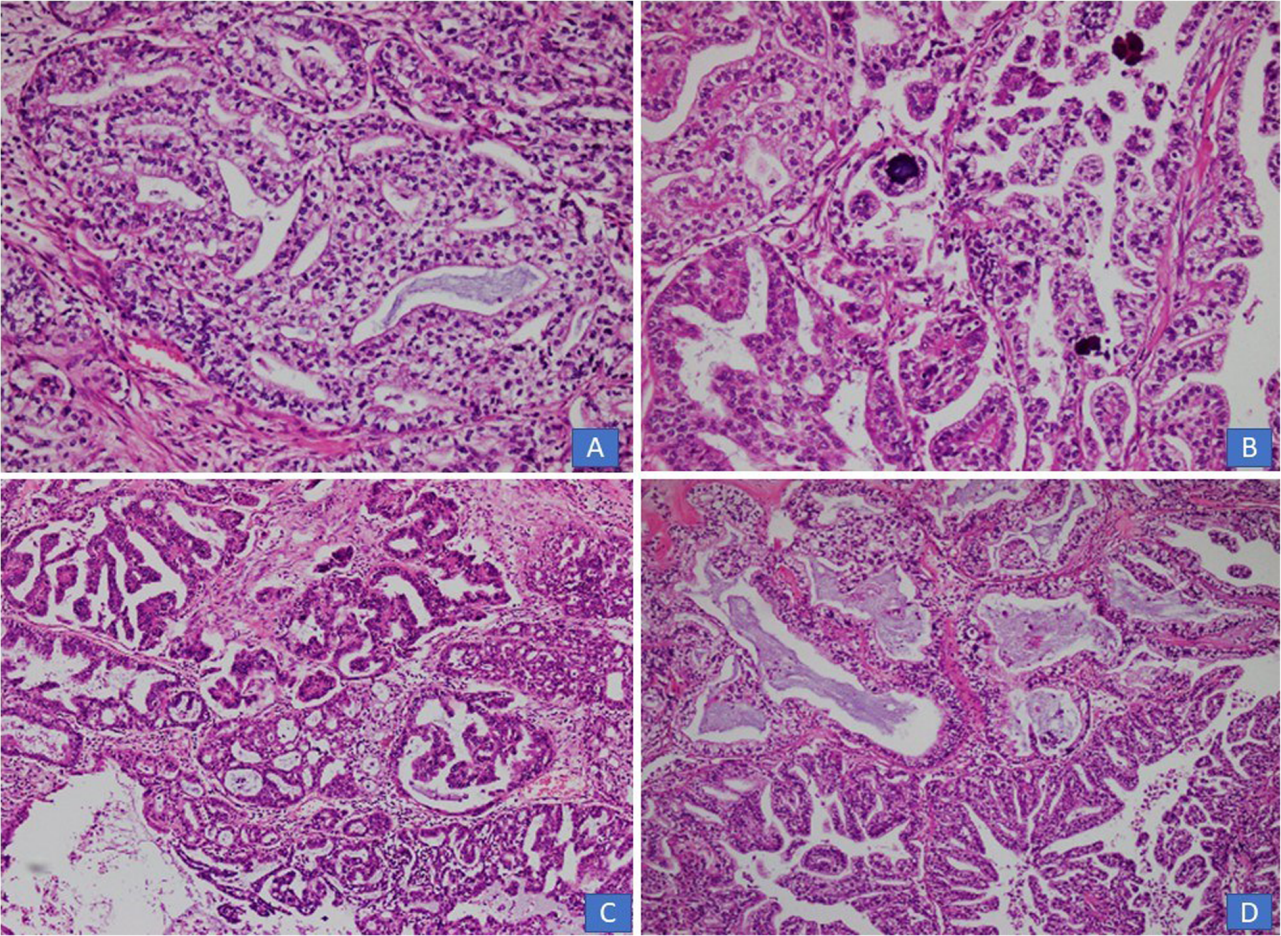
**a** Tumor cells with clear cytoplasm were seen in 2 cases. **b** An area with clear cells, papillary structures and psammomatous calcifications. C&D). Seromucinous carcinoma with stromal invasion

**Fig. 5:**
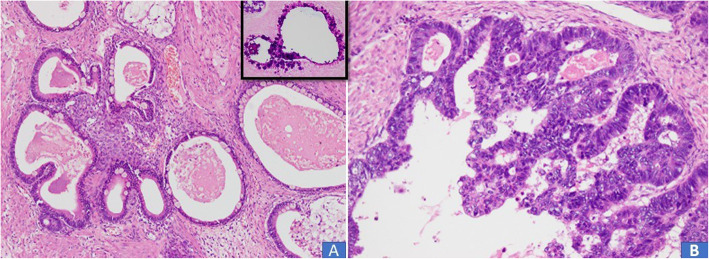
Seromucinous carcinoma recategorized as endometrioid adenocarcinoma with mucinous differentiation. **a** Variably dilated glands lined by columnar epithelium with mucinous cytoplasm. (**Inset**) PAS special stain depicting cytoplasmic mucin. **b** In other areas, high grade tumor was noted with cribriform pattern and pleomorphic columnar lining cells

**Fig. 6:**
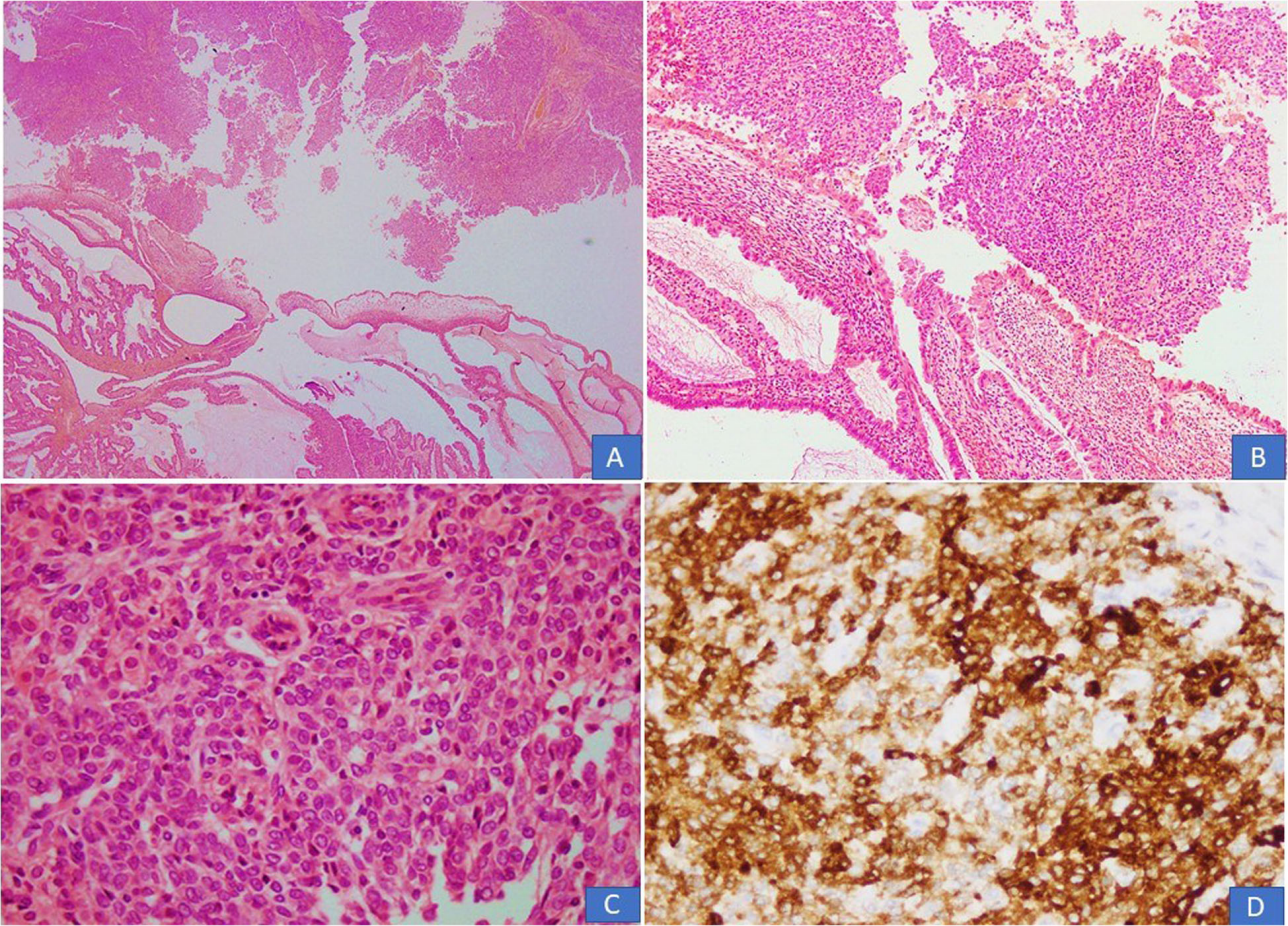
**a**-**c** Seromucinous borderline tumor with co-existent adult granulosa cell tumor. **d** The granulosa tumor cells were positive for Inhibin stain

**Fig. 7:**
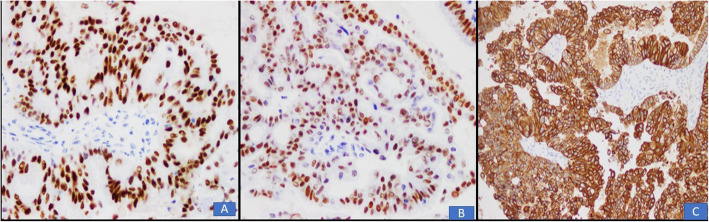
Positive expression of ER (**a**), PAX 8 (**b**) and CK7 (**c**) by tumor cells

In all 10 cases, multiple sections were submitted for histologic examination. On histologic examination, a branching papillary architecture with fibrovascular cores was seen in all 10 cases. The epithelium lining the papillae was stratified and composed of endocervical type mucinous epithelium in some areas and serous epithelium in others **(**Fig. 2a-d). Epithelium was predominantly serous in 7 (70%) cases and predominantly mucinous in 3 (30%) cases. Epithelial cells in all 10 cases demonstrated nuclear pleomorphism and hyperchromasia, irregular nuclear contours, coarse chromatin and occasional prominent nucleoli **(**Fig. 2a-d). Diagnosis of borderline seromucinous tumor was given in 6 cases. A fibromatous stroma was seen in all borderline tumors (Fig. 3a-b). Scattered mitotic figures were seen in all 10 cases. Tumor necrosis was seen in 3 (30%) cases. Clear cells were seen in 3 (30%) cases **(**Fig. 4a, b**)** while squamous epithelium was focally seen in 1 case. Stromal invasion was seen in 4 out of 10 cases resulting in 4 cases being diagnosed as seromucinous carcinoma **(**Fig. 4c, d). However, on reviewing the slides in view of the latest WHO Classification of Female Genital Tract Tumors, all 4 tumors were recategorized as endometrioid carcinomas with mucinous differentiation **(**Fig. 5a, **B** Concomitant endometrioid adenocarcinoma (in contralateral ovary) and concomitant granulosa cell tumor in the same ovary **(**Fig. 6a-d) were seen in 1 case each. In 4 cases, tubal or ovarian endometriosis was noted. The tumors were confined to the ovary in 8 cases. In 1 case with bilateral borderline seromucinous tumor, both fallopian tubes and myometrium were invaded by the tumor while the pelvic lymph nodes, appendix and omentum demonstrated metastatic tumor deposits. Cytoplasmic acid mucin was highlighted in the mucinous cells on special stain PAS ± AB **(**Fig. 5a **inset**). Immunohistochemistry was performed in 7 out of 10 cases. The tumor cells in all 7 cases expressed IHC stains for PAX8, ER and CK7 **(**Fig. 7a, b, c) and PR and were negative for CK20, CDX2 and WT1. In 1 additional case, a right ovarian endometrioid carcinoma with mucinous differentiation, right fallopian tube showed direct tumor invasion while the omentum showed metastatic tumor. Pelvic lymph nodes were received in 3 cases. They were negative in 2 cases and 1 case (described above) showed involvement of lymph nodes by metastatic tumor.

Follow-up was available in 7 cases. One patient with endometrioid carcinoma with mucinous differentiation who had involvement of right tube and omentum (described above) died 18-months postsurgical resection. She had received chemotherapy post resection. Six patients were alive and well at follow up periods ranging from 8 to 46 months. Five of these 6 patients received chemotherapy postsurgical resection (Table 2).

**Table 2:** Details of Follow up (*n* = 7)

S.No	Year resected	Chemotherapy	Length of follow up (months)	Alive	Dead
1	2016	Yes	46	Yes	–
2	2016	No	42	Yes	–
3	2018	Yes	25	Yes	–
4	2018	Yes	18	No	Died Nov, 2019
5	2019	Yes	12	Yes	–
6	2019	Yes	9	Yes	–
7	2019	Yes	8	Yes	–

## Discussion

In a 2016 paper, Kurman and Shih presented a revised and expanded model of ovarian carcinogenesis dividing type I ovarian tumors into 3 groups: (i) endometriosis related tumors (clear cell, endometrioid and seromucinous carcinomas) (ii) low grade serous carcinomas and (iii) mucinous carcinomas and malignant Brenner tumors [7]. Women with endometriosis have been shown to have a two to three-fold increased risk of developing ovarian cancers especially endometrioid and clear cell carcinomas [8, 9]. Recent molecular research has greatly improved our knowledge of various subtypes of ovarian cancers and has an important impact on prognosis and therapy [9].

Seromucinous tumors were first described by Shappell et al. in 2002. They described the clinicopathologic features of 54 ovarian tumors with papillary architecture, predominantly ciliated serous type epithelium as well as endocervical mucinous epithelium. Of their 54 cases, they classified 34 (63%) as atypical proliferative (borderline), 5(9%) as intraepithelial carcinoma, 8 (15%) as microinvasive carcinoma, and 7 (13%) as carcinoma [3]. Although seromucinous tumors were included in the 2014 WHO Classification as a distinct tumor type, a paper by Kurman and Shih published in 2016 again cast doubt on the terminology of seromucinous tumors and questioned whether these tumors truly constituted a distinct type of ovarian tumor. They stressed that morphologic, immunohistochemical and molecular genetic findings show that seromucinous tumors do not show serous-type differentiation and emphasized that the evidence in fact linked these tumors to clear cell and endometrioid tumors. According to the authors, seromucinous tumors are mullerian in origin, characterized by an admixture of various cell types including endocervical-type mucinous, endometrioid and squamous type epithelium and have a close relationship with endometriosis similar to clear cell and endometrioid tumors. Thus, they regarded seromucinous carcinoma as a variant of endometrioid carcinoma. They recommended that these tumors be subcategorized as “mixed mullerian cystadenomas, “mixed mullerian atypical proliferative (borderline) tumors”, and “mixed mullerian carcinomas” [10]. Another recent study by Rambau et al. also concluded that the morphologic diagnosis of seromucinous carcinomas is not very reliable and stressed that these tumors do not express a distinct immunophenotype or genotype. They examined 32 cases which had been diagnosed as seromucinous carcinomas and reported suboptimal interobserver reproducibility for diagnosis of these tumors. They recategorized all 32 cases as endometrioid (23), low grade serous (9) and mucinous (1) carcinomas. They suggested that the category of seromucinous carcinomas be discontinued as with ancillary molecular tests these tumors can be assigned to one of the major tumor categories [11]. In other words, seromucinous carcinoma and endometrioid carcinoma with mucinous differentiation, commonly endocervical like, may show similar and overlapping histological features and their distinction may be arbitrary. In a recent review of ovarian seromucinous tumors, Nagamine and Mikami also recommended that category of seromucinous carcinoma be removed from the classification. According to them, seromucinous carcinomas represent endometrioid carcinomas with mucinous differentiation and should be regarded as a variant of endometrioid carcinoma. According to the authors, seromucinous tumors are borderline in most cases. Nagamine and Mikami as well as Shappell et al. in an earlier study regard high grade nuclear atypia in seromucinous tumors without destructive stromal invasion as “intraepithelial carcinoma” and term frank stromal invasion of < 5 mm in greatest dimension in borderline tumors as “seromucinous borderline tumors with microinvasion” [3, 12]. The 2020 5th Edition of WHO Classification of Female Genital Tumors has removed ‘Seromucinous Carcinoma’ as a distinct entity after first including it in the 2014 4th Edition. It now only recognizes the benign and borderline seromucinous tumors as distinct entities and maintains that ‘Seromucinous carcinoma’ has been removed because it was a poorly reproducible diagnosis and there is significant morphological overlap with endometrioid carcinoma. It asserts that immunohistochemical and molecular studies also suggest that most cases diagnosed as ‘seromucinous carcinoma’ actually represents unusual morphological patterns of endometrioid carcinoma. It now considers seromucinous carcinoma as a subtype of endometrioid carcinoma with mucinous differentiation [5, 6]. There is no doubt regarding the close relationship of seromucinous ovarian tumors with ovarian endometriosis and with other ovarian tumors such as endometrioid and clear cell tumors which also demonstrate a similar close relationship to endometriosis. This is also evident from the fact that endometriosis related ovarian tumors including seromucinous, clear cell and endometrioid tumors show ARID1A mutations with loss of ARID1A expression in a high proportion of cases. Interestingly, a large recent study of endometrioid endometrial carcinoma found a seromucinous component in over 9% of cases and furthermore found that the presence of seromucinous component was associated with a better prognosis in these tumors with a longer progression free survival [13]. Each of these tumors, however, shows additional characteristic molecular alterations which may be increasingly relevant clinically for developing targeted therapies [8–10]. Since seromucinous tumors are very rare, epidemiological data is scanty. However, these tumors have mostly been reported in adults especially in the late reproductive age. Mean and median age in our series of cases were 42 and 41 years respectively. In multiple reported series, mean age of seromucinous carcinomas was 45, 47, and 48 years respectively which are similar to our findings [3, 14, 15]. However, in one recent series, mean age for borderline seromucinous tumors and seromucinous carcinomas was much older-63.2 and 68.3 years respectively [16]. Whereas seromucinous cystadenomas/cystadenofibromas are completely benign, seromucinous borderline tumors also appear to have a good prognosis even in the presence of peritoneal implants. On the other hand, tumors originally diagnosed as advanced stage seromucinous carcinomas appeared to have a bad prognosis although very little data was available [17].

Signs and symptoms in our series were nonspecific and included lower abdominal pain and swelling. Similar nonspecific clinical presentation is reported in literature. In Tang et al’s series of 7 cases, most patients presented with abdominal distension [15]. Radiologically, 7 cases in our series showed multilocular cysts (with focal solid component in 2 cases) while 3 cases showed unilocular cysts. MRI reveals unilocular or multilocular cysts in benign cases while borderline and malignant tumors appear as complex cystic-solid masses [18].

A recent study which looked at the MRI findings of seromucinous tumors found evidence of endometriosis in more than half the cases and concluded that it was difficult to differentiate tumors diagnosed as seromucinous carcinomas from other endometriosis related carcinomas by imaging studies [14]. However, some other recent imaging studies have found certain imaging features on MRI which can help in distinguishing seromucinous tumors from other malignant ovarian tumors including endometriosis related tumors [19, 20].

In our series, tumor laterality was known in 9 out of 10 cases, 5 cases were unilateral and 4 were bilateral. In multiple published series, the majority of cases were unilateral (16 out of 19 in one series, 5 out of 7 in another). In Shappel’s series of 54 cases, 70% were unilateral [3, 14, 15].

Our cases ranged in size from 7 to 35 cm with mean size of 19 cm in the largest dimension. In various published series, tumor sizes ranged from 1.8 to 18 cm with mean size of 9.3 cm, 10.5 cm and 12 cm [3, 14, 15, 17]. According to Nagamine and Mikami, mean size of borderline seromucinous tumors is 8–10 cm [12].

In our series, external surfaces of the cysts were smooth in 9 out of 10 cases while 6 cases demonstrated papillary projections on the inner surfaces. Papillary projections on inner surfaces of cysts in cases reported originally as ovarian seromucinous carcinomas have been reported in multiple series [3, 12, 16].

On histological examination, all 10 cases in our study revealed architectural atypia in the form of branching papillae and a confluent glandular pattern as well as cytological atypia in the form of atypical seromucinous epithelium. Endometriosis was seen in 4 cases. Endometriosis has been noted in 30–70% seromucinous tumors. Mucinous cells are endocervical type. Goblet cells and Paneth cells which are indicators of gastrointestinal differentiation are usually absent and were not seen in any of our cases. Scattered squamous and clear cells were seen in 1 and 2 cases respectively. Of the 10 cases, 4 demonstrated stromal invasion consistent with a diagnosis of seromucinous carcinoma. The histologic features in 3 of these 4 cases corresponded closely with the histologic features of seromucinous carcinomas described in published studies [3, 14, 15, 17]. However, in view of exclusion of seromucinous carcinoma as a distinct entity from the 2020, the 5th Edition of WHO Classification of Female Genital Tract Tumors and its recategorization as Endometrioid carcinoma with mucinous differentiation [5], we recategorized all 4 cases as endometrioid carcinoma with mucinous differentiation on retrospective slide review. In 6 cases, the histological appearance corresponded to the histological features described for seromucinous borderline tumors [3, 14, 15, 17].

Borderline seromucinous tumors and endometrioid carcinoma with mucinous differentiation are usually positive for IHC stains CK7, ER, PR and CA125 as well as PAX8 and are usually negative for CK20, CDX2 and WT1. The positivity for ER and negativity for WT1 is also suggestive of a close relationship with endometrioid and clear cell ovarian tumors [13, 14, 21]. On IHC stains, all our cases demonstrated positivity for CK7, PAX 8, ER and PR and were negative for CK20, CDX2 and WT1. WT1 was negative in areas of serous epithelium also. This is true for seromucinous tumors. The negativity for CK20, CDX2, and WT1 and positivity for ER and PR is useful in routine practice in differentiating borderline seromucinous tumors from borderline serous and mucinous tumors. Serous borderline tumors are almost always positive for WT1 while mucinous borderline tumors are variably positive for CK20 and CDX2 and negative for ER and PR [12]. Thus, our findings were similar to published international data [13, 14, 21]. In 1 of our cases, concomitant endometrioid adenocarcinoma was seen in the other ovary. In another case, concomitant granulosa cell tumor was present in the same ovary. A number of case reports have been published documenting the coexistence of seromucinous borderline tumor and cases originally diagnosed as seromucinous carcinoma of the ovary with other malignant ovarian tumors [22–24].

About 90% borderline seromucinous tumors have FIGO Stage I disease. The remaining show extraovarian disease in the form of peritoneal implants and/or lymph node involvement. Even such patients usually have an excellent prognosis [12, 16]. It must be remembered that the peritoneal implants may be misinterpreted as disseminated adenocarcinoma on frozen sections [12].

We have used the term ‘extraovarian disease’ for the 2 cases which showed direct invasion of one or both fallopian tubes and/or myometrium and metastatic involvement of pelvic lymph nodes, appendix and/or omentum. Although seromucinous carcinomas have now been recategorized as endometrioid carcinomas with mucinous differentiation, the cases originally reported as such demonstrated gross and microscopic features similar to seromucinous borderline tumors including papillary projections, high grade cytologic atypia and architectural complexity. Mitotic index is usually < 5 mitoses/10HPFs. No stage IV seromucinous carcinomas were ever reported, and one 2017 study found that the so called seromucinous carcinomas comprised 4% of all ovarian carcinomas and had a 55% five-year survival rate [17].

Follow up was available in 7 cases. Length of follow up in our study ranged from 8 to 46 months. Chemotherapy was given in 6 out of 7 cases. One out of the 7 patients died. In 6 of our 10 cases, hysterectomy with bilateral salpingo-oophorectomy and in 4 cases cystectomy alone was performed. Nagamine and Mikami believe that patients who have been treated with cystectomy alone, especially those of reproductive age, can be managed conservatively without any further treatment if no residual disease is seen on imaging studies. However, they recommend that such patients should be clearly informed about the risk of recurrence or involvement of contralateral ovary and should be strongly counselled on the need for long term follow up [12].

## Conclusions

In conclusion, out of 10 seromucinous ovarian tumors in our series, 6 were diagnosed as seromucinous borderline tumors and 4 cases originally diagnosed as seromucinous carcinomas were recategorized on slide review as endometrioid carcinomas with mucinous differentiation. Seromucinous ovarian tumors demonstrate relatively special clinicopathological features but often have overlapping morphologic and immunophenotypical features with other ovarian carcinomas including endometrioid carcinomas and low-grade serous carcinomas. However, a panel IHC approach is useful in differentiating them from their histological mimics. Even after their inclusion in the 2014 WHO classification of ovarian tumors as a distinct entity, many authors still believed that it is necessary to investigate their pathogenesis and molecular features even more thoroughly to determine whether these tumors truly represented a distinct category of ovarian tumors or not. The category of seromucinous carcinomas was especially problematic and some authors believed that these actually represented endometrioid carcinomas with mucinous differentiation. They thus advocated its removal from the classification. However, other authors believed that the unique features of seromucinous carcinomas supported their classification as distinct types of ovarian tumors. Finally, the 2020 5th Edition of WHO Classification of Female Genital Tumors reversed Seromucinous carcinoma as a distinct entity recategorizing them as endometrioid carcinoma with mucinous differentiation. The 2020 WHO recognizes only benign and borderline seromucinous tumors. A histogenesis of seromucinous tumors from the secondary Mullerian system or vestigial structures is favored.

## Data Availability

Data and materials of this work are available from the corresponding author on request.

## Abbreviations

WHO: World Health Organization
FIGO: International Federation of Gynecology and Obstetrics
AKUH: Aga Khan University Hospital
ERC: Ethical Review Committee
CK: Cytokeratin
ER: Estrogen Receptor
PR: Progesterone Receptor
WT1: Wilms’ Tumor1
MRI: Magnetic Resonance Imaging
